# Combating powdery mildew: Advances in molecular interactions between *Blumeria graminis* f. sp. *tritici* and wheat

**DOI:** 10.3389/fpls.2022.1102908

**Published:** 2022-12-16

**Authors:** Johannes Mapuranga, Jiaying Chang, Wenxiang Yang

**Affiliations:** College of Plant Protection, Technological Innovation Center for Biological Control of Plant Diseases and Insect Pests of Hebei Province, Hebei Agricultural University, Baoding, China

**Keywords:** wheat, powdery mildew, *Blumeria graminis* f. sp. *tritici*, disease resistance, resistance genes

## Abstract

Wheat powdery mildew caused by a biotrophic fungus *Blumeria graminis* f. sp. *tritici* (*Bgt*), is a widespread airborne disease which continues to threaten global wheat production. One of the most chemical-free and cost-effective approaches for the management of wheat powdery mildew is the exploitation of resistant cultivars. Accumulating evidence has reported that more than 100 powdery mildew resistance genes or alleles mapping to 63 different loci (*Pm1-Pm68*) have been identified from common wheat and its wild relatives, and only a few of them have been cloned so far. However, continuous emergence of new pathogen races with novel degrees of virulence renders wheat resistance genes ineffective. An essential breeding strategy for achieving more durable resistance is the pyramiding of resistance genes into a single genotype. The genetics of host-pathogen interactions integrated with temperature conditions and the interaction between resistance genes and their corresponding pathogen a virulence genes or other resistance genes within the wheat genome determine the expression of resistance genes. Considerable progress has been made in revealing *Bgt* pathogenesis mechanisms, identification of resistance genes and breeding of wheat powdery mildew resistant cultivars. A detailed understanding of the molecular interactions between wheat and *Bgt* will facilitate the development of novel and effective approaches for controlling powdery mildew. This review gives a succinct overview of the molecular basis of interactions between wheat and *Bgt*, and wheat defense mechanisms against *Bgt* infection. It will also unleash the unsung roles of epigenetic processes, autophagy and silicon in wheat resistance to *Bgt*.

## Introduction

Wheat (*Triticum aestivum*, 2n=42; AABBDD) significantly contributes to human calorie consumption, food security and global agricultural sustainability (Godfray et al., 2010). Wheat production demand is driven by the increasing human population; however, it is continuously threatened by various fungal pathogens. Wheat powdery mildew caused by an obligate biotrophic fungal pathogen *Blumeria graminis* f. sp. *tritici* (*Bgt*), is one of the most destructive wheat diseases, and may cause yield losses of 10–40% and, in extreme cases, even up to 50% (Parlange et al., 2015; Zhu et al., 2015; Zhang et al., 2017). The rapid spread and adaptation of the pathogen is enhanced by its short life cycle, the ease with which airborne spores may be spread over long distances, and the possibility of sexual recombination leading to the generation of new virulent races (Jankovics et al., 2015). Powdery mildew infection induces immense transcriptional reprogramming which is strictly regulated by various mechanisms (Buscaill and Rivas, 2014; Jones et al., 2016; Adachi and Tsuda, 2019). Accumulating evidence has reported that histone post-translational modifications and other epigenetic processes, which typically affect the accessibility of DNA to the transcriptional machinery, fine-tune the expression of genes involved in plant defense responses to pathogen infection (Ding and Wang, 2015; Ramirez-Prado et al., 2018; Kong et al., 2020a; Kong et al., 2020b).

Wheat resistance against powdery mildew can either be race-specific or non-race specific resistance. Race-specific resistance imparts full resistance to some particular pathogens, but not others, and it is conferred by single resistance (*R*) (major effect) genes and is relatively heritable. Non-race specific resistance imparts partial resistance which does not rely on specific pathogen avirulence genes and thus allows for infection but reduces pathogen proliferation (Maurya et al., 2021; Mapuranga et al., 2022c). However, pathogens are endowed with exceptional adaptability by long-term survival pressure and environmental cues (Turrà et al., 2014), leading to high pathogen variations (Lei et al., 2017). Since the wheat-*Bgt* interaction is host- or race-specific, new, more virulent races might swiftly evolve and arise, rendering the numerous race-specific resistance genes added in wheat cultivars ineffective in preventing the development of powdery mildew (Kang et al., 2020). Therefore, it is critical to explore new wheat powdery mildew (*Pm*) resistance genes to develop durable prevention and management approaches of this disease. Furthermore, uncovering the underlying regulatory mechanisms of bread wheat defense responses to biotic stresses is crucial for increasing resistance to powdery mildew (Liu et al., 2016). This review gives a critical overview of the molecular basis of interactions between wheat and *Bgt*, and wheat defense response mechanisms to *Bgt* infection, including the role of autophagy and silicon in conferring resistance to powdery mildew. It will also discuss the recent developments in understanding the epigenetic processes and elements such as DNA methylation, and histone modification involved in wheat responses to *Bgt* infection.

## Penetration and colonization in wheat by *Bgt*

The infection strategies and pathogenicity mechanisms of biotrophic fungal plant pathogens including *Bgt* have been extensively reviewed (Chaudhari et al., 2014; Bourras et al., 2015; Bindschedler et al., 2016; Bourras et al., 2016; Bourras et al., 2018; Liang et al., 2018; Sharma and Gautam, 2019; Jaswal et al., 2020; Mapuranga et al., 2022a; Mapuranga et al., 2022b). A prerequisite for compatibility establishment between a pathogen and its hosts is successful penetration. The conidia and ascospores produced by *Bgt* in cleistothecia, are primarily responsible for its propagation, and the disease most often affects a large area after being spread by wind (Jankovics et al., 2015). Following the attachment of conidia or ascospores to the surface of a photosynthetically active wheat leaf, a sophisticated germ tube is produced which then elongates into a thread-like hypha with appressoria (Acevedo-Garcia et al., 2017). Appressoria formation is triggered by the host’s chemical or physical signals, topographic signals, substratum hydrophobicity, and cutin monomers (Jones et al., 2001; Kamakura et al., 2002; Sharma and Gautam, 2019; Chethana et al., 2021). Subsequently, the digitate hypha develops into a penetration peg and ramifies a haustorium in order to facilitate the direct breaching of the host epidermal cell *via* turgor pressure and dynamic enzymes (Glawe, 2008). Subsequently, the fungus will only colonize the cell walls of the epidermal cell layer of leaf tissues. It will then form haustoria, which are the only fungal structures that will grow inside the host tissues, but it will do so without shattering the cell membrane. The haustoria are at the hub of *Bgt* biotrophic interaction through nutrient acquisition from the host and delivering effector proteins into the infected host cells to suppress host immunity (Jankovics et al., 2015). Only a few *Bgt* effectors including AvrPm2, AvrPm3^a2/f^2, AvrPm3^b2/c2^, and SvrPm3^a1/f1^ have been identified and cloned so far (Bourras et al., 2015; Praz et al., 2017; Bourras et al., 2019). These effectors belong to variable effector families and they are all highly induced upon *Bgt* infection to enhance pathogenesis. SvrPm3^a1/f1^ is a ribonucleic effector which suppresses the recognition of AvrPm2, AvrPm3^a2/f2^, AvrPm3^b2/c2^ by their cognate host resistance proteins (Bourras et al., 2019). *Bgt* infection process is also enhanced by mild temperatures (10-22°C). Furthermore, development of conidia that are produced asexually and secondary infections is enhanced by an increase in relative humidity and this will result in polycyclic disease development and many epidemics (Te Beest et al., 2008). Accumulating evidence has reported that wheat surface signals from the cuticle are responsible for inducing the pre-penetration growth of fungal pathogens (Wang et al., 2020; Arya et al., 2021). For instance, knockdown of a cuticle biosynthesis regulator gene, *TaWIN1* reduced cuticle biosynthesis and conidial germination of *Bgt* (Kong and Chang, 2018). Intriguingly, the exogenous application of wax very-long-chain aldehydes, which are absent from the cuticle wax of *TaWIN1*-silenced plants, was able to fully rescue the *Bgt* germination penalty. This suggests that *Bgt* exploits the wax very-long-chain aldehydes biosynthesis which is positively regulated by *TaWIN1* to trigger conidial germination (Kong and Chang, 2018). Consistent with this, the silencing of *TaKCS6* and *TaECR* in bread wheat led to reduced *Bgt* germination and biosynthesis of wax (Wang et al., 2019; Kong et al., 2020b). The use of cuticular wax derived from wild-type wheat plants restored the *Bgt* germination penalty on *TaKCS6*- or *TaECR*-silenced plants (Wang et al., 2019; Kong et al., 2020b). Findings from these studies support the hypothesis that *Bgt* exploits the cuticle biosynthesis genes *TaECR*, *TaWIN1*, and *TaKCS6* as susceptibility genes to enhance its pre-penetration growth.

## Wheat components involved in interactions with *Bgt*

The ability of plants to defend themselves against pathogen infection varies widely, and the interaction outcome is determined by the genetic status of both the pathogen and its host.

### Innate immunity confers wheat resistance to *Bgt*

**Figure 1:**
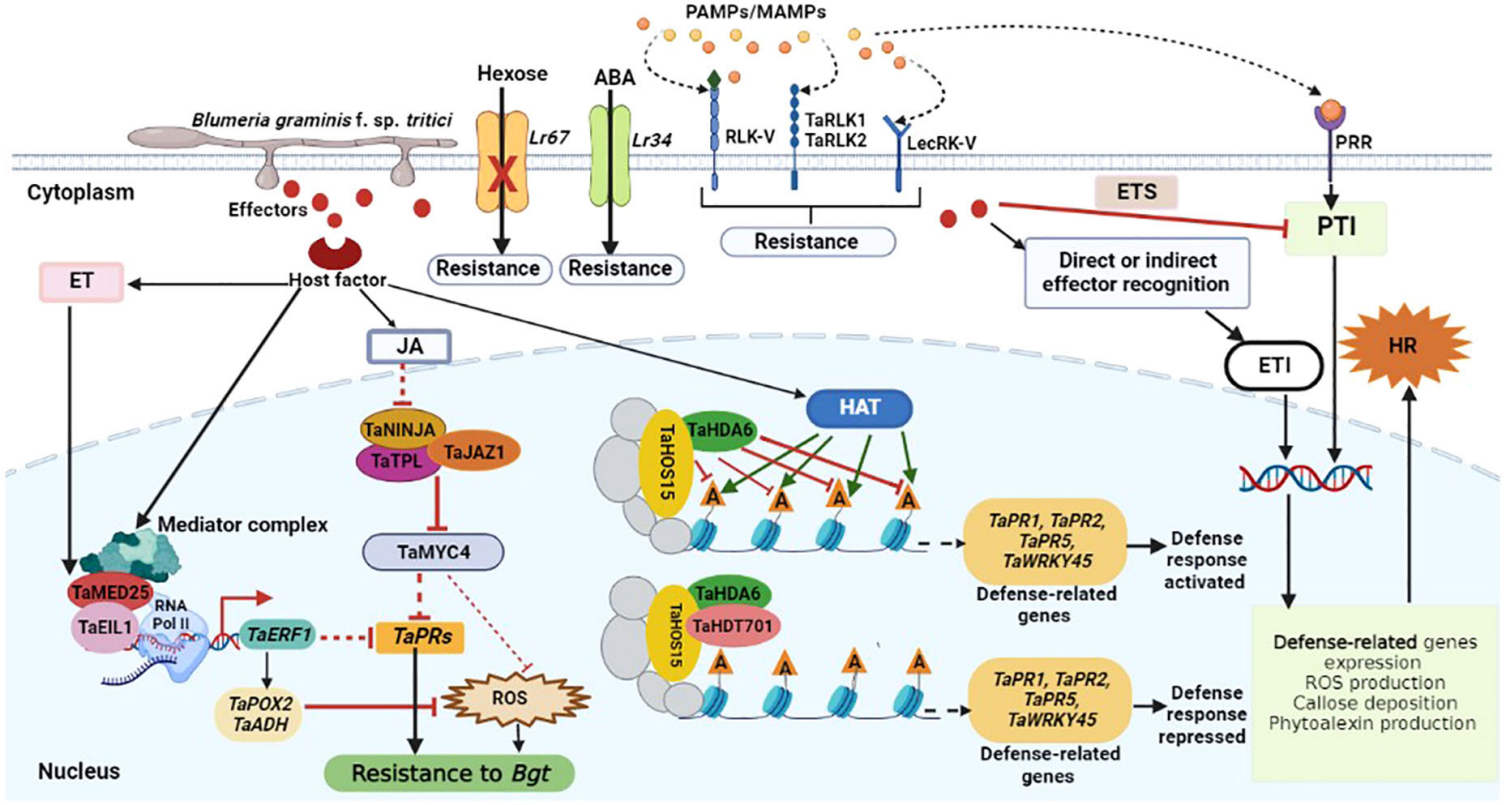
Schematic representation of wheat defense responses to *Bgt* infection. Pathogen-derived conserved molecules (PAMPs/MAMPs) are recognized by pattern recognition receptors (PRRs), and this activates PAMP-triggered immunity (PTI). Pathogens induce susceptibility by interfering with the immune signaling network through the secretion of effectors, resulting in effector-triggered susceptibility (ETS). Following direct or indirect effector recognition, plant R proteins activate host defense responses to inhibit pathogen growth, and this is called effector-triggered immunity (ETI). The receptor-like kinases (*TaRLK1*, *TaRLK2*), malectin-like/LRR-RLK (*RLK-V*), and lectin type RLK (*LecRK-V*) resistance genes, encode proteins that are involved in the perception of fungal determinants and transmission of defense signals to the cell interior. Upon *Bgt*, reactive oxygen species (ROS) and endogenous pathogenesis-related (*PR*) genes are highly induced to enhance defense response to infection. Meanwhile, the physical interaction between TaMED25 and TaEIL1 may lead to the recruitment of RNA Pol II to enhance the transcription of *TaERF1.* TaERF1 may further suppress ROS accumulation and the expression of *PR* genes to partially attenuate wheat basal resistance to *Bgt* infection. TaJAZ1, TaNINJA and TaTPL form a co-repressor complex that suppresses TaMYC4 transcriptional activity under normal conditions. Jasmonate (JA) accumulation in response to *Bgt* infection results in the degradation of TaJAZ1 to release TaMYC4 and other transcriptional factors, consequently leading to the suppression of *TaPRs* expression and ROS accumulation in wheat. Ethylene (ET) also inhibits ROS accumulation and the expression of *TaPRs* to negatively regulate wheat resistance against *Bgt* infection. Furthermore, *Bgt* infection also results in the recruitment of more histone acetyltransferases (HATs) and thus increase histone acetylation at the defense related genes (represented by green arrows), thus activating the expression of these genes. The TaHDT701-TaHDA6-TaHOS15 histone deacetylase complex mediates histone deacetylation at the wheat defense-related genes like *TaPR1*, *TaPR2*, *TaPR5* and *TaWRKY45*, which results in the suppression of defense-related genes transcription and defense responses to *Bgt*.

In order to ward against invading pathogens, plants have developed a multifaceted innate immune system (Dodds et al., 2006). The recognition of pathogen-associated molecular patterns (PAMPs) or microbe-associated molecular patterns (MAMPs) by membrane localized pattern recognition receptors (PRRs) triggers the first layer of innate immunity known at the PAMP-triggered immunity (PTI), which curtails pathogen invasion (Jones and Dangl, 2006; Jones et al., 2016). Downstream effects including mitogen-activated protein kinase (MAPK) pathways activation, defense-responsive genes expression, expression of pathogenesis-related genes, stomatal closure, reactive oxygen species (ROS) burst and callose deposition are triggered by PTI activation (Stael et al., 2015; Jaswal et al., 2020). By secreting effector molecules, pathogens may circumvent PTI and activate effector-triggered susceptibility (ETS), which is then countered by the host’s effector-triggered immunity (ETI), the second layer of innate immunity (**Figure 1**). ETI activation causes a dynamic reprogramming of several defense-responsive genes like MAPK, ROS burst, increase of Ca^2+^ levels, and hormonal change, and is usually accompanied by hypersensitive response and programmed cell death at the infection sites to curb pathogen invasive growth and proliferation (Jones and Dangl, 2006; Dangl et al., 2013; Jones et al., 2016). As proposed initially by Jones and Dangl, the PTI-ETS-ETI cycle continues in a zig-zag model (Jones and Dangl, 2006). Wheat calcium-dependent protein kinases (CDPKs) are critical sensors of calcium concentration changes that occur in response to a variety of biotic and abiotic stresses (Li et al., 2008). *CDPK* genes in wheat respond to a variety of biotic and abiotic stresses, including *Bgt* infection, low temperatures, salt, drought, and abscisic acid. It was found that each *CDPK* gene responded well to several treatments, which lends credence to the notion that wheat *CDPKs* serve as convergence points for many signal transduction pathways (Li et al., 2008). During wheat-*Bgt* interaction, the expression of *CDPK2* was significantly upregulated, hence it was speculated that *CDPK2* plays an essential role in wheat defense responses to *Bgt* infection (Li et al., 2008). Receptor-like kinases (RLKs) which recognize microbial presence in the apoplast, have an extracellular domain, a transmembrane domain and a cytoplasmic kinase domain (Kanyuka and Rudd, 2019). Wheat resistance to powdery mildew was recently found to be positively regulated by *TaRLK1* and *TaRLK2* (Chen et al., 2016), *RLK-V* (Hu et al., 2018) and *LecRK-V* from *Haynaldia villosa*, a diploid wheat relative highly resistant to powdery mildew (**Figure 1**) (Wang et al., 2018). *TaRLK1* and *TaRLK2* are leucine-rich repeat receptor-like kinases (LRR-RLKs), and their ectopic overexpression in wheat resulted in increased ROS accumulation in fungal penetration sites, leading to *Bgt* resistance (Chen et al., 2016). Overexpression of the lectin type RLK gene *LecRK-V* enhanced wheat resistance to *Bgt* (Wang et al., 2018). Wheat basal defense and *Pm21-*mediated resistance to *Bgt* were both found to be regulated by the malectin-like/LRR-RLK gene *LRK-V* (Hu et al., 2018).

*COI1* serves as a key regulator in the jasmonate (JA) signaling pathway. During both compatible and incompatible interactions between wheat and *Bgt, TaCOI1* expression was induced. The rate at which *Bgt* was able to successfully penetrate all *TaCOI1*-silenced Brock was greater, but the proportion of macrocephalic appressoria and resistance responses was lower than in the control group. Based on these results, *TaCOI1* gene positively regulates wheat resistance to *Bgt* infection (Liu et al., 2018). Copines are calcium-dependent phospholipid-binding proteins that are made up of two highly conserved C2 domains at their N-termini, and a von Willebrand factor A (vWA) domain at their C-termini (Creutz et al., 1998). It was found that wheat resistance to *Bgt* is negatively regulated by the wheat copine genes *TaBON1* and *TaBON3*. Both variations in temperature and the presence of pathogens affected the expression of *TaBON1* and *TaBON3*. These findings demonstrated a conserved role of plant copine genes in the plant immunity and revealed new genetic resources for the development of wheat varieties with durable resistance to powdery mildew (Zou et al., 2018a).

The glucan synthase-like (GSL) gene family is found in many plants where it produces callose which is not only present at areas of fungal penetration, but also in callus plugs that form in wounds and in the cells wall of pollen (Voigt et al., 2006). In wheat, a total of eight distinct *TaGSL* genes were found and confirmed to mediate the synthesis and regulation of callose in diverse tissues under distinct environmental cues (Voigt et al., 2006). Recently, Cheng and colleagues showed that expression level of *TaGSL22* was substantially upregulated by *Bgt* infection. Callose deposition was reduced by the silencing of *TaGSL22* which led to increased susceptibility of wheat to *Bgt* (Wang et al., 2022b). Therefore, *TaGSL22* positively regulates wheat resistance to *Bgt*. Genes known as Recognition of *Peronospora parasitica* 13-like, (*RPP13-like*) genes which are members of the NBS-LRR superfamily serve important roles in the resistance of various plant species to many pathogens. It was recently established that wheat resistance to powdery mildew is positively regulated by *TaRPP13L1-3D* gene *via* a RanGAP-WPP complex-mediated nucleocytoplasmic pathway (Zhang et al., 2022). These fresh insights into *TaRPP13L1-3D* will be beneficial in dissecting the methods by which wheat develops its resistance to biotrophic fungal pathogens and understanding the link between *R* gene expression and pathogen defense. Generally, different degrees of plant resistance to a particular pathogen species are dependent on how the host interacts with the pathogen, either *via* broad-spectrum (non-race specific resistance) or race-specific (specific resistance to a single race) resistance.

### Race-specific resistance

Race-specific resistance is also called qualitative resistance, all stage resistance or monogenic resistance, and is generally mediated by a single major *R* gene, which becomes active upon detection of the pathogen’s effectors in a gene-for-gene paradigm (Maurya et al., 2021; Mapuranga et al., 2022c). Although wheat powdery mildew resistance genes have been shown to activate the PTI and ETI pathways, the nature of this activation has remained unclear. Due to the rapid evolution of new pathogen races, major *R* genes are known to deteriorate easily, which results in a short lifetime for resistance. This results in a restricted window of opportunity for protection (McDonald and Linde, 2002). To determine which resistance genes are ineffective, it is necessary to know the identification of the race-specific resistance genes present in wheat cultivars. Molecular marker assisted selective breeding is an efficient and rapid approach for identifying resistance genes. Wheat disease resistance genes have been mapped with the use of molecular markers. Recent research into *Pm* genes has made extensive use of simple sequence repeat (SSR) and single-nucleotide polymorphism (SNP) markers (Wu et al., 2019).

The longevity of race-specific resistance genes in the field may be extended by the use of gene combinations, strategic gene deployment, and multiline cultivars (Burdon et al., 2014; Li et al., 2014; Mundt, 2018). However, there is a tendency of many pathogen isolates to coexist in their respective ecological habitats. As a consequence of genetic variety in the pathogen population, this may increase the probability of a disease epidemic. Because of this, it’s possible that gene stacking won’t be enough to halt the spread of disease for very long, and the cultivars will lose their efficacy. In order to increase the efficacy of gene stacking, allelic mining was proposed for the improvement of agriculture (Bhullar et al., 2010). Accumulating evidence has reported that multi-allelic race specific resistance genes evolve under intense diversifying selection to detect specific pathogen avirulence alleles (Bhuiyan et al., 2009; Kanzaki et al., 2012). For example, at the *Pm3* locus, race-specific resistance is conferred by 17 functional alleles (Brunner et al., 2010). *Pm3* encodes a CC-NBS-LRR protein, with complete sequence conservation in the CC domain across these alleles, but sequence exchange is shown in the NBS and LRR sections, suggesting gene conversion or intragenic recombination between the alleles (Bourras et al., 2015). Using map-based cloning of haploid F_1_ populations, some studies identified *Bgt* effectors *AvrPm2*, *AvrPm3^a2/f^2*, *AvrPm3^b2/c2^*, and *SvrPm3^a1/f1^* corresponding to some NLR resistance genes in wheat (Bourras et al., 2015; Praz et al., 2017; Bourras et al., 2019). Bourras and colleagues characterized the corresponding Avr loci of Pm3 alleles and proposed the involvement of a pathogen-encoded suppressor of avirulence (Svr) in race specificity (Bourras et al., 2015). It was found that the function of detected avirulence alleles is blocked by an active suppressor, resulting in a disease. The presence of Svr in the powdery mildew will, in combination with the avirulence allele and the R allele, regulate plant resistance in the wheat-powdery mildew pathosystem. This suggests that there are additional layers of a simple gene for the gene model controlling *Avr-R* interactions in the wheat-powdery mildew system. A recent study on the AvrPm3-Pm3 model, further demonstrated the importance of Pm3 NLR receptors in concurrently determining host-specificity for grass mildews and race-specific resistance to wheat powdery mildew (Bourras et al., 2019). *Pm12* and *Pm21* have recently been found to be orthologous genes (Zhu et al., 2022). Distinct intramolecular interaction patterns and race specificities in the two orthologous resistance genes *Pm12* and *Pm21* were clearly demonstrated (Zhu et al., 2022). Multiple alleles at the *Pm2* locus have been discovered across a wide range of genotypes (Jin et al., 2018). Powdery mildew resistance genes *Pm2a*, *Pm2b* and *PmCH1357* were mapped to the same genomic level, and cloning of these genes using different approaches revealed that they genes confer different levels of resistance, suggesting that it might be imparted by distinct resistance genes or alleles (Sánchez-Martín et al., 2016; Chen et al., 2019a; Jin et al., 2022).

### Non-race specific resistance

Non-race specific resistance is also called quantitative resistance, minor gene resistance or adult plant resistance. This type of resistance often manifests as a partial resistance phenotype, in which pathogen invasive growth and proliferation is suppressed without any immune response overtly shown (Maurya et al., 2021; Mapuranga et al., 2022c). It is also called minor gene resistance because just a few genes are responsible for imparting the resistance (Maurya et al., 2021). Because it provides partial levels of resistance and field tolerance, it is also known as standard/partial/field resistance. Adult stages of wheat development is associated with the manifestation of this resistance, and is hence regarded as adult plant resistance (Li et al., 2014). Most of the identified *Pm* genes are race specific all-stage resistance genes, and only a few pleiotropic partial resistance genes, such as *Pm38/Yr18/Lr34/Sr57/Lt1* (Singh et al., 2012), *Lr46/Yr29/Pm39/Sr58/Ltn2* (Singh et al., 2013) and *Pm46/Yr46/Lr67/Sr55/Ltn3* (Herrera-Foessel et al., 2014), which confer partial resistance to powdery mildew and other rust pathogens at the adult plant stage, hence they are adult plant resistance genes. Isolation of adult plant resistance genes *Pm38* and *Pm46* found a single gene encoding an ATP-Binding Cassette (ABC) transporter (Krattinger et al., 2009) and a hexose transporter (Moore et al., 2015) at the multi-pathogen resistance locus, respectively, which confers a dual resistance for wheat leaf rust and stripe rust in addition to resistance to powdery mildew (**Figure 1**). It was suggested that these two genes would go through a defense activation process associated with a coordinated interaction between abscisic acid and sugar signaling (Moore et al., 2015; Krattinger et al., 2019). These sugar signals may operate as priming molecules that lead to PTI and ETI (Bolouri Moghaddam and Van den Ende, 2012), modulating plant metabolism that is induced by the fungal pathogen. The activation of sugar transporters is necessary for upsetting the carbohydrates equilibrium between sources and sinks, which is often seen in response to biotic stresses (Valluru and Van den Ende, 2011). Accumulating evidence has established that non-race specific resistance is more robust and durable than race-specific resistance in combating the new emerging virulent pathogen races (Liu et al., 2001; Li et al., 2014). For example, after 20 years of cultivation, the cultivar Knox has shown consistent adult plant resistance effectiveness to powdery mildew (Liu et al., 2001). In particular, breeders are interested in powdery mildew adult plant resistance genes that confer resistance to a wide range of pathogens.

## Cloning and characterization of wheat powdery mildew resistance genes

The first powdery mildew resistance gene in wheat was found in wheat variety ‘Thew’ by an Australian researcher Waterhouse in 1930 (Zeller, 1973). Since then, new *Pm* genes have been identified from common wheat and wheat relatives. Although more than 100 powdery mildew resistance genes/alleles in 63 loci (*Pm1*-*Pm66*) have been documented (McIntosh et al., 2019; Zhang et al., 2019), only a few of them have been cloned and characterized so far (**Table 1**) including *Pm1a* (Hewitt et al., 2021), *Pm2a* (Sánchez-Martín et al., 2016), *Pm2b* (Jin et al., 2022), *Pm3b* (Yahiaoui et al., 2004), *Pm5e* (Xie et al., 2020), *Pm8* (Hurni et al., 2013), *Pm17* (Singh et al., 2018), *Pm21* (Xing et al., 2018; He et al., 2018), *Pm24* (Lu et al., 2020), *Pm38* (Krattinger et al., 2009), *Pm41* (Li et al., 2020), *Pm46* (Moore et al., 2015), and *Pm60* (Zou et al., 2018b). The integration of the high-quality wheat genome sequence (IWGSC Ref-Seq v1.0) (Avni et al., 2017; Appels et al., 2018; Maccaferri et al., 2019), and various advanced molecular cloning approaches for reducing the complexity of the genome to allow the performance of targeted resequencing analyses has facilitated the cloning of wheat *Pm* genes. Some of these genes including *Pm1a* (Hewitt et al., 2021), *Pm2a* (Sánchez-Martín et al., 2016; Manser et al., 2021), and *Pm4b* (Sánchez-Martín et al., 2021), were cloned using the Target-sequence Enrichment Sequencing (TEnSeq) pipelines. TEnSeq pipelines include various approaches such as Mutagenesis and Resistance gene enrichment Sequencing (MutRenSeq), Mutagenesis Chromosome flow sorting and short-read Sequencing (MutChromSeq), Association genetics with Resistance gene enrichment Sequencing (AgRenSeq) and Targeted Chromosome-based Cloning *via* long-range Assembly (TACCA). AgRenSeq and MutRenSeq are based on NLR-targeted DNA capture and hybridization, while MutChromSeq and TACCA are based on purification of individual chromosomes from wheat lines (Zhang et al., 2020). The majority of the powdery mildew resistance genes were cloned using the map-based cloning approach (Yahiaoui et al., 2004; Krattinger et al., 2009; Yahiaoui et al., 2009; Moore et al., 2015; Sánchez-Martín et al., 2016; Zou et al., 2018b; Li et al., 2020; Lu et al., 2020; Hewitt et al., 2021), with a few having been cloned using the homology-based cloning approach (Hurni et al., 2013; Singh et al., 2018).

**Table 1:** A summary of cloned wheat powdery mildew resistance genes.

Gene cloned	Origin	Chromosome position	*R*-gene product	*R*-gene class	Cloning Technique	References
*Pm1a*	*T. aestivum*	7AL	A CC-NBS-LRR protein of 964 amino acid residues	ASR	Map-based cloning, MutChromSeq	(Hewitt et al., 2021)
*Pm2a*	*T. aestivum*	5DS	A CC-NBS-LRR protein of 1 277 amino acid residues	ASR	MutChromSeq	(Sánchez-Martín et al., 2016; Manser et al., 2021)
*Pm2b*	*T. aestivum*	5DS	A CC-NBS-LRR protein of 1 277 amino acid residues	ASR	Map-based cloning	(Jin et al., 2022)
*Pm3a, b*	*T. aestivum*	1AS	A CC-NBS-LRR protein of 1 415 amino acid residues	ASR	Map-based cloning	(Yahiaoui et al., 2004; Srichumpa et al., 2005)
*Pm3c, 3CS, d, e, g & k*	*T. aestivum*	1AS	A CC-NBS-LRR protein of 1 413 amino acid residues	ASR	Map-based cloning	(Srichumpa et al., 2005; Yahiaoui et al., 2006; Yahiaoui et al., 2009)
*Pm3f*	*T. aestivum*	1AS	A CC-NBS-LRR protein of 1 414 amino acid residues	ASR	Map-based cloning	(Srichumpa et al., 2005)
*Pm4b*	*T. aestivum*	2AL	MCTP-kinase of 746 amino acid residues, a putative chimeric protein of serine/threonine kinase and multiple C2 domains and transmembrane regions	ASR	MutChromSeq	(Sánchez-Martín et al., 2021)
*Pm5e*	*T. aestivum*	7BL	A CC-NBS-LRR protein of 1 067 amino acid residues	ASR	Map-based cloning	(Xie et al., 2020)
*Pm8*	*Secale cereale*	1RS	A CC-NBS-LRR protein of 1 375 amino acid residues	ASR	Homology-based cloning	(Hurni et al., 2013)
*Pm17*	*Secale cereale*	1RS	A CC-NBS-LRR protein of 1 414 amino acid residues	ASR	Homology-based cloning	(Singh et al., 2018)
*Pm21*	*Dasypyrum villosum*	6VS	A CC-NBS-LRR^**^ protein of 908 amino acid residues	ASR	Map-based cloning, MutRenSeq	(He et al., 2018; Xing et al., 2018)
*Pm24*	*T. aestivum*	1DS	A Tandem kinase protein of 893 amino acid residues with putative kinase-pseudokinase domains	ASR	Map-based cloning	(Lu et al., 2020)
*Lr34/Yr18/Sr57/Pm38/Ltn1*	*T. aestivum*	7DS	A 1 401 amino acid ABC transporter	APR	Map-based cloning	(Krattinger et al., 2009; Krattinger et al., 2019)
*Pm41*	*T. turgidum* spp. *dicoccoides*	3BL	A CC-NBS-LRR protein of 984 amino acid residues	ASR	Map-based cloning	(Li et al., 2020)
*Lr67/Yr46/Sr55/Pm46*	*T. aestivum*	4DL	A 514 amino acid hexose transporter with 12 trans-membrane helices	APR	Map-based cloning	(Moore et al., 2015)
*Pm60, b*	*T. urartu*	7AL	A CC-NBS-LRR protein of 1 534 amino acid residues	ASR	Map-based cloning	(Zou et al., 2018b; Zou et al., 2022)
*Pm60a*	*T. urartu*	7AL	A CC-NBS-LRR protein of 1 374 amino acid residues	ASR	Map-based cloning	(Zou et al., 2018b; Zou et al., 2022)
*PmR1*	*T. urartu*	7AL	A CC-NBS-LRR protein of 1 218 amino acid residues	ASR	Map-based cloning	(Zou et al., 2018b)
*MlIW172*	*T. urartu*	7AL	A CC-NBS-LRR protein of 1 454 amino acid residues	ASR	Map-based cloning	(Wu et al., 2022)

T. aestivum – Triticum aestivum, CC-NBS-LRR – coiled coil nucleotide binding site leucine-rich repeat, ASR – all-stage resistance, APR – adult plant resistance, ABC – ATP-binding cassette

**An earlier report found that Pm21 was Sr/Thr kinase (Cao et al., 2011), but two subsequent studies found that it is a NLR.

Except for *Pm24*, *Pm38*, and *Pm46*, which encode tandem kinase gene WTK3, an ABC transporter, and a hexose transporter, respectively, most of the cloned powdery mildew resistance genes in wheat encode NLR proteins which are race-specific (**Table 1**). In recent years, tandem kinase protein (TKP) has emerged as a novel kinase protein family in plant innate immunity. A serine/threonine non-arginine-aspartate (non-RD) receptor-like protein kinase with two putative tandem kinase domains (Kin I and Kin II) and lacking an extracellular domain is encoded by the Chinese wheat landrace Hulutou’s *Pm24* (WTK3) gene. A rare 6-bp natural deletion of lysine-glycine codons was found to be endemic to wheat landraces in Shaanxi Province, China, in the kinase I domain (Kin I) of WTK3, which is essential for the gain of function of powdery mildew disease resistance (Lu et al., 2020). The isolation of WTK3 with the tandem kinase domains laid a foundation for comprehension of the signal transduction function of TKP in wheat powdery mildew resistance and plant innate immunity. Understanding the molecular mechanisms of this protein family’s role in plant innate immunity will be enhanced by the characterization of the TKP kinase functions, related proteins, and phosphorylation processes. The cloning of powdery mildew resistance genes paves a way for the development of high-throughput diagnostic functional markers that can be utilized in markers-assisted selection for fungal-resistance breeding programmes.

## The network of wheat resistance to *Bgt*

The network of resistance can be analyzed using transcriptomics (gene regulation and expression profiling), proteomics (protein identification and effects), and metabolomics (metabolite profiling, regulation, pathway and intermediates). However, there are no metabolic profiling studies that have been reported so far during wheat-*Bgt* interactions, hence this section will specifically focus on transcriptomics and proteomics.

### Transcriptomics

Expression and regulation of genes, as well as the identification of important genes involved in the stress response pathway, may all be understood by transcriptome profiling. Depending on the amount of available genomic resources generated and the type of plant being studied, different methods including RNA-sequencing, Affymetrix GeneChips, and spotted micro arrays, expressed sequence tags, sequencing in conjunction with suppression subtractive hybridization, are employed to study the transcriptome. RNA-Seq surpassed other transcriptomics methods because it is the most high-throughput, cost-effective and efficient method due to recent developments in next-generation sequencing technology. Transcriptomics research has so far been extensively conducted in wheat. Transcriptional and translational alterations of plants are also triggered by pathogen attack and this results in the activations of several genes and metabolic pathways as a defense mechanism. Epidermal *Bgt* infection in diploid wheat (*Triticum monococcum*) triggered the expression of 12 genes involved in methyl unit biosynthesis and supply, showing that genes involved in methyl unit production pathways are also important for the host defense response (Bhuiyan et al., 2007). An mRNA tag analysis was performed to explore the cellular metabolic processes that are activated by H_2_O_2_ treatment in wheat. It was found that 260 transcripts had conserved expression across all three wheat lines and these genes might represent a subset of basal H_2_O_2_ sensitive genes involved in signal transduction, cell defense, redox homeostasis, photosynthesis, lipid metabolism, glucose metabolism, and transport (Li et al., 2011).

Another comparative transcriptome analysis of wheat in response to *Bgt* infection established an approximately equal gene expression level in both resistant and susceptible cultivars before *Bgt* inoculation (Xin et al., 2012). Various pathways such as flavonoid biosynthesis, cell wall fortification and metabolic processes were found to be involved in wheat defense responses to powdery mildew (Xin et al., 2012). SGT1 is responsible for the positive regulation of many plant *R* genes that confer race-specific resistance (Muskett and Parker, 2003; Azevedo et al., 2006). The expression of *SGT1* was shown to have a very close relationship with the expression of *R* genes, the hypersensitive response, and the activation of specific resistance proteins (Bhaskar et al., 2008; Meldau et al., 2011). *Haynaldia villosa Hv-SGT1* transcript levels were substantially increased by *Bgt* infection. Transcript levels of *Hv-SGT1* were also substantially upregulated by H_2_O_2_ and methyl jasmonate treatments (MeJA). However, these levels were only minimally stimulated following exposure to ethephon or abscisic acid; but were not changed by salicylic acid (SA) treatment (Xing et al., 2013). As a result, the involvement of *Hv-SGT1* in the production of H_2_O_2_ correlates with the hypersensitive response as well as jasmonic acid signaling. This innovative demonstration that wheat with overexpressed *Hv-SGT1* had higher resistance to powdery mildew, implies that it might serve as a transgenic genetic resource in wheat breeding for the purpose of producing wheat cultivars with durable resistance to multiple pathogens (Xing et al., 2013).

Powdery mildew infection also resulted in differential expression of wheat genes in which 289 transcripts were upregulated and 154 transcripts were downregulated during *R*-gene mediated incompatible interactions between wheat and *Bgt* (Li et al., 2013). Another comparative transcriptome analysis also revealed different activations in wheat’s response to powdery mildew and stripe rust infections (Zhang et al., 2014). In *Bgt-*infected leaves, the differentially expressed genes were grouped into seven pathways, whereas in *Pst-*infected leaves they were grouped into four enriched Kyto Encyclopedia of Genes and Genomes (KEGG) pathways. Many genes and pathways were activated in the wheat-pathogen interaction pathways in response to *Bgt* infection than in response to *Pst* infection (Zhang et al., 2014). Liu et al. analyzed the transcriptomic changes in wheat response to *Bgt* infection in a continuous time period and their findings confirmed involvement of *CMPG1-V*, which encodes the U-box E3 ubiquitin ligase, in augmentation of broad-spectrum resistance against powdery mildew (Liu et al., 2020). A weighted gene co-expression network analysis revealed important *CMPG1-V* regulatory candidates including glucosyltransferase in flavonoid biosynthesis, serine/threonine-protein kinase in phosphorylation, peroxidase in oxidative stress, and defense factor WRKYs (Liu et al., 2020). This study laid a foundation for the elucidation of *CMPG1-V* resistant regulatory network and the identification and molecular characterization of the key candidates that might be exploited in resistant breeding programs.

Another recent transcriptomics study demonstrated that the gene(s) located at 2M^b^ in the Chinese Spring (CS)-*Aegilops biuncialis* 2M^b^ disomic addition line TA7733 imparted a high degree of resistance *Bgt*. When compared to CS, there were 7 278 identified unigenes that displayed unique expression in TA7733 both pre-and post-*Bgt* infection. It was found that 53 out of the 295 unigenes genes encoded NLR proteins and were unique to 2M^b^ and had the potential to be involved in powdery mildew resistance. The establishment of TA7733 broad-spectrum resistance to *Bgt* and availability of putative candidate *R*-gene specific molecular markers derived from 2M^b^ laid a foundation for transferring powdery mildew resistance from 2M^b^ to common wheat by inducing CS-*Ae. biuncialis* homoeologous recombination (Li et al., 2019). Using bulked segregant RNA-Seq, Ma and colleagues analyzed 2 973 differentially expressed genes to study resistance gene regulatory genes. They found that after *Bgt* invasion, six disease-related genes had unique expression patterns, making them prime candidates for elucidating resistance mechanisms and enhancing long-term resistance in wheat to powdery mildew (Ma et al., 2021). Glutathione S-transferase (GST), epitomizes a group of ubiquitous proteins found in plants that comprise several functional proteins. A lot of studies indicated that GSTs are involved in secondary metabolism (Mueller et al., 2000), growth and development (Gong et al., 2005), and biotic and abiotic stress responses in plants (Bianchi et al., 2002). The presence of GST in the leaves of emmer wheat leads not only to a reduction in oxidative stress but also to an enhancement in the plant’s resistance to herbicides (Karpenko et al., 2019). In common wheat, a comprehensive genome-wide analysis uncovered 346 *GST* genes and 87 *TaGSTU* family members. During wheat-*Bgt* incompatible interaction, *TaGSTU6*, *TaGSTU4*, and *TaGSTU7* genes were are highly upregulated (Hao et al., 2021).

### Proteomics

Proteomics is the study of the structural and functional features of all of the proteins present in a live organism at the same time. It can be performed using various approaches including, two-dimensional (2-D) gel electrophoresis, western blotting, matrix-assisted laser desorption ionization-time of flight (MALDI-TOF), mass spectrometry, and enzyme-linked immunosorbent assay (ELISA), together with several bioinformatic tools (Baggerman et al., 2005; Gevaert et al., 2011; Chaudhary et al., 2019). Recent advances in the field of proteomics have made it possible to conduct high-throughput proteome analyses. The majority of proteomic studies has been done on crops including wheat, rice, barley, maize, potato, and soybeans since these crops are the only ones whose whole genome sequences are accessible in the public domain (Yadav et al., 2022). A comparative proteomic analysis of wheat’s response to *Bgt* infection in wheat *Pm30* near-isogenic found 97 differentially expressed proteins and 26 of them belonged to a variety of functional categories, including energy and metabolism, disease and defense, transcription and translation, and signal transduction (Wang et al., 2012). Another proteomic analysis of wheat defense response to *Bgt* also established that 44 differentially expressed proteins were related to the defensive response, photosynthesis, metabolism, and other cellular activities in wheat (Mandal et al., 2014). Quantitative proteomic analysis of N9134, a resistant wheat line, identified 394 protein-species that showed differential accumulation (Fu et al., 2016). These differentially accumulated protein-species predominantly consisted of oxidative stress responsive proteins, pathogenesis-related polypeptides, and primary metabolic pathway components (Fu et al., 2016). MAPKs are involved in the regulation of a variety of plant processes, such as metabolism and the signaling processes involved in abiotic and biotic responses (Gawroński et al., 2014). During incompatible wheat-*Bgt* interaction, MAPK5 protein level was substantially upregulated, suggesting that it serves an essential role in wheat resistance to *Bgt* (Li et al., 2017b).

Liang and colleagues analyzed the proteome of three distinct wheat cultivars at three different time intervals and found that various proteins were upregulated and downregulated in response to *Bgt* infection in wheat cultivar with *Pm40* gene (Liang et al., 2019). It was inferred that the proteins have the capacity to mediate the immune responses and coordinate various physiological and cellular processes that take place during wheat defense response to *Bgt* infection (Liang et al., 2019). Recently a quantitative proteomic analysis revealed that TaGSTU6/TaCBSX3 interaction serves a crucial role in wheat resistance to *Bgt* infection (Wang et al., 2022a). Seven hundred and forty-one differentially accumulated proteins were identified and intriguingly, 42 of them responded to both *Bgt* and *Pst* infection. These findings imply that wheat has distinct mechanisms of responding to *Pst* and *Bgt* infections, since the interaction of TaGSTU6 with TaCBSX3 only imparts resistance to *Bgt* (Wang et al., 2022a). Altogether, these proteomics studies provide a new basis for broadening our understanding of the molecular mechanism of the wheat-*Bgt* interactions at the protein level, as well as a theoretical framework for resistance breeding and the long-term management of powdery mildew.

## Transcriptional regulation of wheat’s defense response to *Bgt*

Although accumulating evidence has reported the transcriptional regulation of wheat’s defense response to biotic stresses; the transcriptional regulation of wheat resistance to *Bgt* infection remains to be explored. The molecular and functional characterization of transcription factors can be exploited in wheat breeding programs for the development of durable broad-spectrum resistance in wheat cultivars. Three transcription factors namely, T16.17353, T4.32876, and T19.62870, belonging to the TGA family of bZIP were found to be involved in wheat resistance to *Bgt* infection (Zhang et al., 2014). Furthermore, there was significant upregulation of *WRKY33* gene orthologs T13.35253 and T16.5876 during wheat-*Bgt* interactions and they were enriched in the plant-pathogen interaction pathway (Zhang et al., 2014). This clearly demonstrated the involvement of WRKY33 transcription factor in the defense response of wheat to *Bgt* infection through the PTI (Mapuranga et al., 2022c). Eukaryotes have a conserved multisubunit complex called mediator that enhances transcription by bridging certain transcription factors with RNA polymerase II (Chadick and Asturias, 2005). The MED25 Mediator component has been studied in *Arabidopsis* for its role in regulating plant defensive responses through interactions with particular transcription factors involved in plant hormone signaling (Kidd et al., 2009; Chen et al., 2012). Wheat’s resistance to powdery mildew was found to be negatively regulated by the TaMED25-TaEIL1-TaERF1 signaling module (Liu et al., 2016). It was demonstrated that *TaMED25* promotes *TaEIL1*-mediated activation of the powdery mildew-responsive gene *TaERF1*. It was also shown that the TaMED25-TaEIL1-TaERF1 signaling module represses the expression of particular pathogenesis-related genes (*TaPRs*) and prevents ROS accumulation in bread wheat leaf cells to fine-tune resistance to powdery mildew. Both *TaMED25* and *TaEIL1* negatively regulated wheat response to *Bgt.* Pol II can be recruited to the promoter of *TaEIL1* and target gene *TaERF1* by the interaction between TaMED25 and TaEIL1, which further suppresses ROS accumulation by upregulating the expression of ROS-scavenging genes (**Figure 1**) (Liu et al., 2016). Zhou and colleagues established that, overexpression of wheat NAC transcription factor *TaNAC6-A* enhances resistance to *Bgt*, but resistance against *Bgt* was compromised by the silencing of the *TaNAC6s* (Zhou et al., 2018). This implies that wheat *TaNAC6s* are essential for the positive regulation of wheat broad-spectrum resistance to *Bgt*. Moreover, it was also suggested that wheat *TaNAC6s* might be regulated by JA, and this feedback regulation of the JA pathway increases resistance against *Bgt* infection. Therefore, wheat *TaNAC6s* share a common signaling pathway with their orthologs *HvNAC6* and *ATAF1* to confer resistance to powdery mildew (Zhou et al., 2018).

Accumulating evidence has reported the involvement of JA in the regulation of wheat responses to abiotic and biotic stresses (Cao et al., 2014; Zhao et al., 2014; Xu et al., 2016; Li et al., 2017a). Based on wheat genome data, Wang and colleagues found 14 *JAZ* (Jasmonate-ZIM domain) genes and demonstrated that the transcription of some of these *JAZ* genes was affected by a wide range of abiotic stress treatments and phytohormones (Wang et al., 2017). Overexpression of a truncated *TaJAZ1* without the Jas motif was demonstrated to enhance the expression of *TaPR1/2* gene and accumulation of ROS in transgenic bread wheat lines, thereby increasing resistance to powdery mildew. Based on these observations, it seems that there is a synergistic action of the JA and ET signaling pathways to regulate the resistance of wheat against powdery mildew, most likely *via* the modification of the common downstream processes of ROS accumulation and expression of pathogenesis-related genes (Jing et al., 2019). The transcriptional activity of TaMYC4, a JA-induced bHLH transcription factor was repressed by TaJAZ1 *via* direct interaction (**Figure 1**). While the N-terminal EAR motif of TaNINJA was shown to interact with the transcriptional co-repressor TaTPL, the ZIM domain interacted with the C-terminus of TANINJA. These results collectively demonstrated that *TaJAZ1* positively regulates wheat resistance to *Bgt* infection (Jing et al., 2019). To increase bread wheat’s resistance to powdery mildew, scientists may utilize genome-editing tools like CRISPR/Cas9 to generate gain-of-function mutations by modifying the Jas motif of specific *TaJAZ* genes like *TaJAZ1.* The *TaJAZ* genes have the potential to be used in molecular breeding, but how they affect the susceptibility/resistance of wheat against other biotrophic fungal pathogens other than *Bgt* remains to be figured out. The expression of *Pm2b* was highly upregulated by *Bgt* infection isolate E09. Self-association of *Pm2b* through its NB domain was also detected. Intriguingly, it was found that *Pm2b* interacts with TaWRKY76-D and TaWRKY76-D silencing showed negatively regulated wheat resistance to powdery mildew (Jin et al., 2022). *Pm2b* probably interacted with TaWRKY76-D to alleviate TaWRKY76D’s suppression of downstream genes. *Pm2* has been shown to either directly or indirectly interact with its avirulence gene AVRPM2 when infected with *Bgt* isolates, resulting in strong hypersensitive response (Praz et al., 2017). Therefore, it was suggested that the interaction between Pm2 and TaWRKY76-D was enhanced by AVRPM2, leading to improved powdery mildew resistance in wheat. Likewise, the recognition of PM2 by AVRPM2 could be facilitated by WRKY76-D, *via* interaction with Pm2 and this will initiate a defense response to infection. Nevertheless, *TaWRKY76-D* silencing failed to confer complete resistance to *Bgt* infection, suggesting that TaWRKY76-D may serve a crucial role in the PM2-mediated powdery mildew resistance pathway (Jin et al., 2022).

## Epigenetic processes in wheat defense response to *Bgt* infection

Epigenetics refers to heritable gene expression alterations that occur between DNA and its surrounding chromatin without changing its DNA sequence and resulting in substantial changes in any organisms’ genome (Kong et al., 2020a). Several studies reported the direct involvement of epigenetic processes and elements in the transcriptional reprogramming and regulation of defense response genes (Deleris et al., 2016; Kim et al., 2017; Chen et al., 2019b), and in establishing memory to environmental cues (He and Li, 2018; Ashapkin et al., 2020). Therefore, such responses are influenced by both the host’s epigenetic configuration and the effects of biotrophic interactions (Ramos-Cruz et al., 2021). DNA methylation, histone modifications, and chromatin remodeling are the major components of the epigenetic make up of plants. Local chromatin accessibility is dictated by single or combined epigenetic marks, which in turn regulates gene expression and, thus may play a role in plant defense, and other processes (Ramos-Cruz et al., 2021).

### DNA methylation

DNA methylation is a type of DNA chemical modification that regulates DNA stability, chromatin structure and even gene expression without altering the nucleotide sequence (Kong et al., 2020a). In plants, cytosine methylation is detected in the context of CG, CHG, and CHH (where H is any nucleotide except G) (Henderson and Jacobsen, 2007; Law and Jacobsen, 2010), in which CG is the most plentiful and common methylation site (Kong et al., 2020a). DOMAINS REARRANGED METHYLTRANSFERASE 2 (DRM2) is the enzyme that catalyzes plant *de novo* methylation *via* the RNA-directed DNA methylation (RdDM) pathway which is a plant-specific pathway (Matzke and Mosher, 2014; Kong et al., 2020a). DNA methylation is important for both transcriptional gene silencing and preservation of genomic integrity by silencing transposable elements. Several studies have recently reported the significance of DNA methylation in plant defense responses to abiotic and biotic stresses (Dowen et al., 2012; Yu et al., 2013; Le et al., 2014; Deng et al., 2017). DNA methylation, and CHH methylation to be specific, has recently been linked to the regulation of defense responses to *Bgt* infection in *Aegilops tauschii* (Geng et al., 2019). There was a significant downregulation of ARGONAUTE4a (AGO4a) in *A. tauschii* following *Bgt* infection. This was accompanied by a significant reduction in the amounts of AGO4a-sorted 24-nt siRNA, particularly for genes near transposable elements (TGAs). The downregulation of AGO4a and 24-nt siRNAs suggests the presence of an active defense mechanism in *A. tauschii* that coordinately downregulates DNA methylation, thereby upregulating the expression of defense-related genes. The differential expression of 24-nt siRNAs between compatible and incompatible interactions with *Bgt* supports the hypothesis that siRNAs are key players in basal defense responses by directing DNA methylation. The presence of many differentially methylated regions (DMRs) was associated with hypomethylation (Geng et al., 2019). Furthermore, it was established that TGAs with CHH-hypomethylated DMRs were enriched in genes for stress response functions including the receptor kinases, pathogenesis-related genes and peroxidases, indicating the involvement of DNA methylation in the regulation of wheat responses to powdery mildew infection (Geng et al., 2019). The upregulation of a pathogenesis-related β-1,3-glucanase gene in response to *Bgt* infection exemplified the effect of CHH-hypomethylation. These results support the hypothesis that dynamic DNA methylation constitutes a regulatory layer within the intricate mechanisms of plant immunity, that may be targeted in an effort to enhance wheat resistance against powdery mildew. However, more research is required to determine whether *Bgt*-derived sRNAs downregulate *AGO4a* and decrease the total siRNA or CHH methylation levels during wheat-*Bgt* interactions (Geng et al., 2019).

### Histone modification

Chromatin modifications and remodeling have been reported to be involved in the transcriptional reprogramming and regulation of defense related genes in plants (Ding and Wang, 2015; Espinas et al., 2016). Histones in chromatin can be subjected to a variety of covalent modifications, including methylation, acetylation, ubiquitination, and phosphorylation. These modifications can change the structure of the chromatin so that it is either permissive or repressive to transcription by modifying interactions between DNA and the histones (Jenuwein and Allis, 2001). The acetylation of histone Lys residues catalyzed by histone acetyltransferases (HATs), can relax the chromatin structure and promote transcription, whereas the process of chromatin compaction involving removal of the acetyl group, which is induced by histone deacetylases (HDACs), is associated with transcriptional repression (Kurdistani and Grunstein, 2003). Recently, various HDACs have been reported to serve as positive and negative regulators of plant defense response to pathogen infections (Ding and Wang, 2015; Ramirez-Prado et al., 2018; Kong et al., 2020a). Scaffolding proteins, also known as proteins with WD repeats (WDRs), often act as platforms onto which protein complexes may be assembled, indicating that they are involved in various cellular processes including secondary metabolism, defense responses, and abiotic stress tolerance (Guerriero et al., 2015).

Transcription factors have been demonstrated as key players in the recruitment of chromatin-remodeling and histone modification machinery to key gene promoters (Luo et al., 2012; Jing et al., 2013). TaHDA6, a wheat RPD3 type deacetylase interacted with TaHOS15, a WDR protein, to inhibit expression of defense-related genes *via* histone deacetylation, thus negatively regulating wheat response to *Bgt* (**Figure 1**) (Liu et al., 2019). It was found that both *TaHDA6* and *TaHOS15* can bind to promoters of defense-related genes. Nevertheless, *TaHDA6* binding ability to these promoters was eliminated when *TaHOS15* was silenced, suggesting that *TaHOS15* directs *TAHDA6* to these promoters for histone deacetylation. These findings laid a foundation for future studies to further identify the transcription factors associated with *TaHOS15* and characterize their roles in the TaHOS15-TaHDA6 HDAC complex recruitment to actual promoters (Liu et al., 2019). The wheat histone deacetylase TaHDT701, a member of the histone deacetylase 2 (HD2), is involved in the regulation of wheat resistance to *Bgt* infection. Recently, it was found that TaHDT701 interacts with TaHDA6 and TaHOS15 to form a histone deacetylase complex. It was shown that the deacetylase complex TaHDT701-TaHDA6-TaHOS15 negatively regulates wheat resistance against powdery mildew by altering the chromatin state of defense-related genes such as *TaWRKY45*, *TaPR1*, *TaPR2*, and *TaPR5* (**Figure 1**). The expression of *WRRKY45*, *TaPR1*, *TaPR2* and *TaPR5* was significantly upregulated by the knockdown of *TaHDT701*, *TaHDA6* and *TaHOS15*. This was accompanied by increased histone acetylation, methylation and reduced nucleosome occupancy at the promoters of these genes, suggesting that the histone deacetylase complex TaDHT701-TaDHA6-TaHOS15 negatively regulates wheat resistance to *Bgt* by modifying altering chromatin state of defense related genes (**Figure 1** **)** (Zhi et al., 2020). This was the first study to show that the histone deacetylase complex HD2-RPD3-WD40 has a role in controlling plant defense responses to pathogen infection.

### Chromatin remodeling

The process of chromatin remodeling, which alters the accessibility of particular DNA sections to transcription machinery may also play a role in regulating chromatin structure and gene expression (Narlikar et al., 2013; Chen et al., 2017). It was recently demonstrated that wheat CHD-type chromatin remodeling factor TaCHR729 interacts with the TaKPAB1, a bHLH transcription factor which recognizes the E-box cis element in the TaKCS6 promoter, resulting in the transcriptional activation of *TaKCS6*, a wax biosynthesis gene (Wang et al., 2019). Intriguingly, it was found that TaCHR729 enhances H3K4me at *TaKCS6* promoter region, thereby promoting *TaKCS6* transcription, and positively regulates the biosynthesis of wheat cuticular wax. Silencing of the expression of *TaKCS6* and *TaKPAB1* downregulated wax accumulation, demonstrating that wheat cuticular accumulation might be positively regulated by the transactivation of *TaKCS6* by TaKPAB1 (Wang et al., 2019). The TaGCN5-TaADA2 module, in association with TaEPBM1, mediates histone acetylation in the TaECR promoter region (Kong et al., 2020b). *TaGCN5* and *TaADA2* knockdown downregulated the expression of *TaECR* and significantly reduced wax accumulation, signifying that wheat wax biosynthesis is triggered by the epigenetic activation of *TaECR* caused by the TaGCN5-TaADA2 histone acetyltransferase complex (Kong et al., 2020b). Similarly, it was also demonstrated that *TaCHR729* knockdown reduced cuticular wax biosynthesis and *Bgt* conidia germination, suggesting that wheat chromatin remodeling factor TaCHR729 mediates histone methylation and fine-tunes cuticular wax biosynthesis to regulate the interaction between wheat and *Bgt* (Wang et al., 2019). The findings from these studies clearly demonstrate that various epigenetic regulators like the histone modifying enzymes and chromatin remodeling elements epigenetically regulate wheat cuticle biosynthesis gene expression.

## Autophagy differentially regulates wheat defense response to *Bgt*

Autophagy, also known as self-eating, is a process whereby cell organelles and cytosolic macromolecules are digested in lysosomes (or vacuoles in plants and yeast) in order to recycle nutrients (Liu and Bassham, 2012; Li and Vierstra, 2012). In plants, the process of autophagy is responsible for both “pro-death” and “pro-survival” functions in regulating programmed cell death associated with ETI. Autophagy contributes to defense responses during plant-biotrophic pathogen interactions (Hofius et al., 2009; Minina et al., 2014). Autophagy-related proteins ATG4 and ATG8 are essential for the biogenesis of autophagy. In the early stages of the *Pm21*- and *Pm3f*-triggered wheat incompatible responses to *Bgt*, two times of transcript accumulation of ATG4s/ATG8s were detected. The first transcript accumulation was detected during early stages of infection and the second one was detected during late stages of infection. Fluorescence microscopy also revealed a *Bgt*-induced upregulated wheat autophagy level in the *Pm21*-triggered incompatible interaction. *Bgt*-induced transcript accumulation of ATG4s/ATG8s was also observed in the late stages of infection of a susceptible line isogenic to the *Pm21* resistance line although it was not much higher than that in incompatible interactions. These findings showed that ATG4/ATG8-associated autophagy in the early stage and late stage positively and negatively regulates wheat immune responses to *Bgt*, respectively (Pei et al., 2014). Furthermore, the expression of wheat ATG4s/ATG8s was shown to be elevated by abiotic stress factors and typically regulated by various phytohormones (Pei et al., 2014). ATG6 proteins are pleiotropic proteins that serve in autophagy as well as the phosphatidylinositol 3-phosphate signaling pathways. Wheat ATG6s are involved in powdery mildew immunity, in which they positively regulate *Pm21*-triggered resistance response and negatively regulate basal resistance in susceptible plants. Silencing of the ATG-heterologous protein in wheat inhibited the broad-spectrum resisted conferred by *Pm21*, demonstrating that autophagy positively regulates wheat resistance to *Bgt* (Yue et al., 2015). The inevitable arrest of successful conidia penetration on knockdown plants before developing into large mycelia suggests that TaATG6 serves a weak positive function in the *Pm21*-triggered resistance response, most likely in the early stages of *Bgt* invasion. This implies that, the dynamics of host-pathogen interactions are influenced by autophagy-mediated defense responses. *Bgt* targets the autophagy process in its host cells because it remobilizes cellular nutrients which it may exploit to accomplish the nutritional supply for its invasive growth and proliferation.

## Silicon regulates wheat resistance against *Bgt* infection

**Figure 2:**
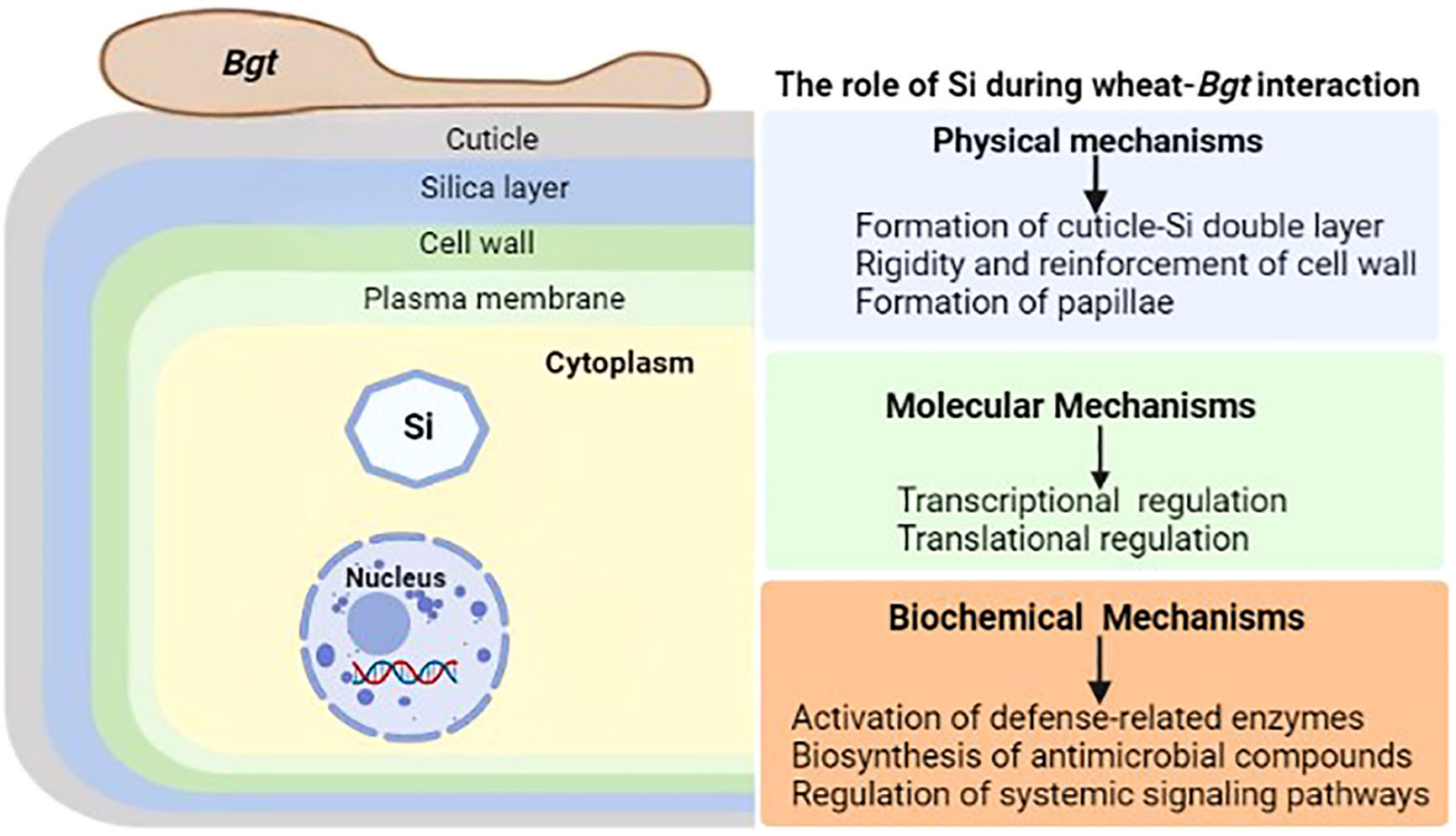
General illustration of the role of silicon (Si) during wheat-*Bgt* interactions. Wheat defense responses mediated by Si are generally classified into physical, molecular and biochemical mechanisms. Physical mechanisms involve the formation of cuticle-Si double layer, reinforcement of cell wall and papillae formation. Molecular mechanisms involve the transcriptional and translational regulation of key defense-related genes and proteins. Biochemical mechanisms are attributed to the activation of defense-related enzymes, stimulation of antimicrobial compounds biosynthesis and regulation of the complex systemic network of signaling pathways.

Silicon (Si) is the second most common element in the Earth’s crust, yet its importance in biota is still up for debate (Exley, 1998). It is considered as a quasi-essential bioactive element not important for the growth and development of plants (Epstein, 1972), but accumulating evidence has reported its significant involvement in plant defense responses to abiotic and biotic stresses. Initial speculation about Si’s protective role focused on its ability to strengthen the cell wall, for example, against the invasion of fungal hyphae, but subsequent research has shown that Si’s action on plants is far more complex, involving a cross-talk with the cell interior and an effect on the metabolic processes of the plant (Luyckx et al., 2017). Physical barriers, such as wax, cuticle, and cell-wall protection, and post-formed defensive barriers, such as cell-wall reinforcement and papillae deposition at infection sites, are the basis for the resistance induced by Si against a broad array of pathogens. During plant-pathogens interactions, Si induces or reinforces biochemical or molecular mechanisms to enhance plant resistance against biotic stresses. This includes the activation of defense-related compounds like phytoalexins, phenolics and momilactones and defense-related enzymes like polyphenol oxidase, peroxidase, phenylalanine ammonia lyase and lipoxygenase (Rémus-Borel et al., 2005; Rahman et al., 2015). The unleashing of the unsung roles of silicon in wheat resistance to powdery mildew will benefit many disciplines including agriculture, and ecology (**Figure 2**).

Plants exposed to Si form a silica layer in between cuticle and cell wall, which serves as a physical barrier and promotes plant resistance to pathogenic fungal infection (Islam et al., 2020). It was established that Si can mitigate the effects of various wheat fungal diseases including powdery mildew by modifying their monocyclic components like infection efficiency, incubation time, quantity of lesions per unit area of infected leaf, lesion size, and the rate at which the lesion expands (Pazdiora et al., 2018). Both physical barriers and metabolic defenses act synergistically to reduce disease severity (Debona et al., 2017). Si deposition below the cuticle and an increase in papillae formation at infection sites have been attributed to the physical barrier in wheat (Bélanger et al., 2003; Filha et al., 2011). Si also increased defense enzyme activity, biosynthesis of phytoalexins and phenolic compounds, and H_2_O_2_ accumulation at the infection sites, thus priming the biochemical defense responses (Pazdiora et al., 2021). The presence of a phenolic-like material associated with degraded powdery mildew haustoria was observed in epidermal cells of Si-treated wheat leaves infected with *Bgt* (Bélanger et al., 2003). Fungitoxic aglycones were found in different concentrations in plants that were treated with Si compared to the control. In wheat plants that were not treated with Si, *Bgt* formed a haustorium that was well developed, whereas in Si-treated plants osmiophilic deposits remained behind (Bélanger et al., 2003). Furthermore, collapsed conidial chains and intense fluorescence was observed in *Bgt*-infected wheat leaves at the sections that were treated with Si. These findings strongly suggest that wheat plants treated with Si are able to produce phytoalexins in response to *Bgt* infection, that is, active localized defense responses to *Bgt* infection are mediated by Si (Bélanger et al., 2003; Rémus-Borel et al., 2005). Si can bind to hydroxyl groups of proteins strategically involved in signal transduction or interfere with cationic co-factors of enzymes regulating pathogenesis-related genes, acting as a modulator affecting the timing and degree of plant defense responses, the same way secondary messengers induce systemic resistance. As a result, Si may induce resistance by interacting with several factors within plant stress signaling systems (Fauteux et al., 2005).

A transcriptomic study revealed minimal indication of regulation of a particular metabolic activity, and the response to the supply of Si on control (uninfected) plants was restricted to 47 genes of various functions (Chain et al., 2009). *Bgt* inoculation led to an upregulation of a plethora of stress- and metabolism-related genes while simultaneously downregulating the expression of genes involved in photosynthesis. Si-treatment to *Bgt*-infected plants halted the spread of disease and resulted in an almost complete reversal of the genes controlled by only *Bgt* effects (Chain et al., 2009). These findings imply that Si has a little effect on a plant’s transcriptome in the absence of stress, even in the case of a monocot that accumulates a lot of Si, like wheat. Conversely, the transcriptome alterations generated by *Bgt* were counteracted by the addition of Si, which had the added advantage of reducing the impact of biotic stress (Chain et al., 2009). About 900 genes reacting to pathogen infection were changed in control leaves of wheat-*Bgt* infected plants, whereas the pathogen altered very few genes in Si-treated plants, indicating that the stress caused by *Bgt* invasion was almost eliminated by Si (Chain et al., 2009). As a result, Si appears to minimize the effect of pathogen infection on the transcriptome of host plants, most probably by limiting the exploitation of pathogen virulence factors, rather than promoting resistance *via* transcriptional reprogramming of defense-related genes (Van Bockhaven et al., 2015). Si can induce the formation of a thicker cellulose membrane, whereas the severity of the disease in wheat in the field can be reduced by the double cuticle layer, density of long and short silicified plant epidermal cells, thick silica layer below the cuticle thereby curbing yield losses (Pozza et al., 2015). It was also established that, as the Si content in the culture medium increased, the pathogen index decreased, the grain mass and number together with the dry weight of shoots increased significantly (Moldes et al., 2016). Multivariant analysis revealed that an increase in Si treatments significantly reduced the antioxidant activities of some biochemical parameters such as superoxide dismutase, catalase, and ascorbate peroxidase. Furthermore, increase in Si doses resulted in a decrease in *Bgt* proliferation in foliar surfaces. It was concluded that an increase in Si concentration and reduced activity of antioxidant enzymes is closely associated with a reduction of powdery mildew in wheat (Moldes et al., 2016). Incorporating the Si functions in reduction of plant stresses into development of molecular methods can now be facilitated by recent advances in genomics and metabolomics. It has been shown that Si-application reduces biotic stress over time, which may be useful for ecologically integrated strategies that aim to improve resistance of crops to biotic stresses and boost yield without resorting to pesticides.

## Conclusion and future perspectives

Undoubtedly, wheat powdery mildew is among the most destructive diseases that are threatening global wheat production. To combat this disease, breeders can utilize genetically resistant cultivars, which is both cost-effective and chemical-free. As genomics of agricultural plants and their linked wild species improves, the gene pool available for enhancing powdery mildew resistance may be increased and widely utilized. Future breeding programs should aim to find exploited and unexploited *Pm* genes, and the introgression of these genes, to develop a large number of pathogen-resistant cultivars. Characterizing wheat germplasm at the pathological level, followed by molecular characterization utilizing linked markers for known *Pm* genes, would be one of the most efficient approaches for combating the expanding problem of biotic stresses in wheat. Classical breeding has been successful in amassing resistance, and cultivars with reasonable levels of resistance have been identified by germplasm screenings. Resistance characterization and efficient application in wheat breeding have been enhanced by the adoption of modern phenotyping methodologies, the extraordinary growth of genetic resources, and the introduction of speed breeding tools.

Despite the recent technological and scientific advancements in the management of wheat disease, plant pathogens continue to threaten global wheat production. Climate change may directly or indirectly affect fungal pathogens and their respective diseases they cause. Factors such as rainfall, temperature, relative humidity, wind speed and direction, affect the pathogenesis, invasive growth, proliferation, spread and the ultimate survival of the pathogen. The most significant factors in determining the success or failure of a specific host-pathogen interaction, as well as the spread and survival of the pathogen are temperature, relative humidity and precipitation. Therefore, before investigating the effects of climate on powdery mildew development, it is of great importance to predict the possible repercussions of the climate change on the host, pathogen, their interaction, population dynamics, micro-evolutionary developments and the structure of the agro-system community. Powdery mildew can therefore be effectively managed by harnessing genetic resistance and integrated powdery mildew management methods to develop more durable wheat resistant cultivars.

## Author contributions

Conceptualization, JM; literature search, JM; writing—original draft preparation, JM; writing—review and editing, JM, JC, and WY; funding acquisition, WY. All authors contributed to the article and approved the submitted version.
